# Construction of Garden Landscape Design System Based on Multimodal Intelligent Computing and Deep Neural Network

**DOI:** 10.1155/2022/8332180

**Published:** 2022-07-07

**Authors:** Xueyong Yu, Heng Yu, Chunjing Liu

**Affiliations:** ^1^Liaocheng University, Shandong, Liaocheng 252000, China; ^2^Sangmyung University, Seoul 03016, Republic of Korea

## Abstract

The problem of module discrimination and identification in the field of landscape design is the focus of researchers. Based on multimodal intelligent computing, this paper constructs a landscape design system based on deep neural network. The article first uses a deep neural network to train multimodal garden landscape images, and then performs pooling and convolution operations on garden landscape images on the multimodal training model of convergence speed on the edge and solve the problem of low model accuracy. In the simulation process, the neural network module of MATLAB software is used to extract the spatiotemporal features of the dynamic garden landscape image from the three directions of the bottom block of the garden to achieve feature complementarity. This method only uses 15% of the features of the original feature set. The complexity of the recognition system also reduces the recognition error rate. The experimental results show that by adopting the design of feature series fusion, maximum value fusion, and multiplicative fusion in the score layer, the feature series fusion achieves a high accuracy rate under the multiplicative fusion of the three modalities, reaching 77.1%, and the test error is within 0.118, which effectively improves the multimodal characteristics of the integrated landscape and makes the modeling results more accurate.

## 1. Introduction

Ecological gardens refer to gardens designed according to the principles of ecology. Through the differences of natural environment, ecological landscape types with diverse colors and unique regional characteristics are constructed [1]. The garden landscape static model aims to recommend the minimum cost for the network before submitting the computing task to the multimodal network, according to the deadline and workload of the computing task, and the number of computing resources that can ensure the task is completed on time [2–4]. Then, the dynamic model will monitor the running status of the task in real time during the execution of the task and dynamically adjust the number of computing resources when the running speed cannot meet the deadline requirement and finally ensure that the computing task can be completed on time. Traditional methods based on hand-built features are not robust, while training of deep neural network interfaces on small datasets will result in severe overfitting [5–7].

At present, most of the elastic resource management systems provided by multimodal network service providers are based on thresholds for resource quantity control. Such systems rely on the network itself to configure these thresholds to decide when to adjust the amount of computing resources [8–11]. For example, the network needs to set the CPU usage threshold of leased virtual machines. When the CPU usage of virtual machines exceeds this threshold, the multimodal network platform will automatically add new virtual machines to the computing cluster. In the experiments, the accuracy of the classifier exceeds that of the previous classifier. Usually, the prediction of the completion time of MapReduce tasks requires a detailed analysis of the entire computing process. However, a MapReduce job consists of several distinct phases, each of which will have different computational resource requirements. At the same time, there are dependencies between different stages [12–14].

Based on the above background, this paper proposes a garden image design method based on audio-visual information fusion, which combines high-order multimodal garden landscape image features with garden image features multimodal feature fusion, which improves the robustness of the garden image model accuracy. First, a large-scale continuous garden image is designed and the equipment is used to record the garden landscape image data. Then, the multimodal garden landscape image features and garden image features of different dimensions are selected through experiments and multimodal feature fusion is carried out. Finally, the garden image model modeling, training, and decoding of DNN-HMM is carried out on the platform, the garden landscape data is preprocessed, and the multimodal features of the garden landscape are extracted, respectively. In order to realize the fusion of multimodal features, this paper proposes a multimodal deep fusion method based on deep neural network combined with categorical cross-entropy loss to learn high-level fusion features of garden landscapes. The weights are extracted and the feature screening criteria are defined according to the network learning characteristics and the difference in activation weights of each type of feature is calculated and compared to obtain a dimensionality reduction and efficient speech emotion cognitive feature set F. On this basis, based on B/S architecture, using Vue.js, Spring boot, MySQL, and other technologies, this paper designs and implements a garden landscape recommendation system based on multimodal features.

## 2. Related Work

Ecological gardens with perfect landscape design are the perfect combination of natural beauty and artificial beauty and are composed of different plant spaces, shapes, and color changes to achieve a beautiful landscape higher than nature. The research on multimodal neural network mainly focuses on the representation of multimodal data features and the correlation mechanism between modalities. Among them, the research on multimodal data feature representation mainly focuses on the representation of text data and the representation of landscape image data [15–17]. Among them, methods such as word2vec can take each independent word as a feature and map it into a feature vector. At the same time, the semantically related words have greater similarity between the mapped feature vectors. For garden landscape image data, the commonly used methods for extracting features include gradient direction histogram features.

With the widespread use of neural networks, Peter et al. [18] believe that the use of neural networks for feature extraction has become a more general choice. Unsupervised learning, such as autoencoder and deep belief network (DBN) can learn the representation of landscape images in low-dimensional space from landscape image data. In order to schedule tasks on this task flow, Karterouli and Batsaki [19] developed a set of resource management strategies based on constraint programming. At the same time, Dai et al.[20] also designed a resource management algorithm that can sense whether the data to be processed is local, to ensure that the entire system can meet the service quality requirements. We extracted all eight categories of sentiment data for experimental testing, and the average recognition error rate of the baseline category was reduced by 2.1% when the DCNN classifier was constructed using the entire feature set. Resource management systems like Jockey and CRESP also use a static model. These works use different approaches, Jacob et al, [21] constructed a quantitative relationship between task run setting parameters and completion time for estimating the amount of computing resources that need to be reserved.

Yahia et al. [22] used the CSP algorithm to extract features from electrical signals, and the extracted feature matrix contains a lot of redundant information. In order to improve the speed of network training, reduce the complexity of the system, and further improve the design rate, it is necessary to feature the filter. Deep neural network is an excellent design method because it can automatically extract features during the training process, so it can be effectively applied to some problems. However, current research only focuses on the operation results of deep networks, ignoring the analysis of the nature of network behavior [23]. Therefore, paying attention to the learning behavior of the neural network itself and observing the learning process of the entire network to the feature is a problem worthy of research.

## 3. Multimodal Intelligent Computing and Deep Neural Network Cascade

### 3.1. Multimodal Hierarchical Sorting Operation

When the size of the multimodal network training set matches the network scale, the trained network can effectively extract the features of the input garden images. If the data in the small data set is similar to the data in the large data set (such as photos of real objects), the network can also effectively extract the characteristics of garden images on the new data set. At this time, the dimension of the data has been effectively reduced, and a new classification layer can be added after the output of the network for classification.

(1) $$\begin{array}{c} f\left(x,x\left(t\right)\right)=\max imumv\left\{\left(x\left(t\right),t\right),\left(x^{2}\left(t\right),t\right),\left(x^{3}\left(t\right),t\right),\ldots ,\left(x^{n}\left(t\right),t\right)\right\}. \end{array}$$

The front part of the multimodal network is composed of 5 network interface modules connected in sequence. The front-end network interface accepts three-channel multimodal garden landscape images with a resolution of 224 × 224 as input. In each module, there are 2 or 3 network interface layers consisting of 3 × 3 network scores, which are connected end to end and whose outputs are activated using the ReLU function.

(2) $$\begin{array}{c} q\left(i,j\right)-\frac{\text{delta}\left[x\left(i,j\right)-x\left(j-1\right)\right]}{\sum_{i,j<t}x\left(i,j\right)-x\left(i-1\right)}-\frac{\text{delta}\left[x\left(i,j\right)+x\left(j+1\right)\right]}{\sum_{i,j<t}x\left(i,j\right)+x\left(i-1\right)}=0. \end{array}$$

A garden landscape is input, after data preprocessing, feature extraction, and feature fusion, the cosine similarity between the input garden landscape and the garden landscape in the database is calculated, the obtained similarity is sorted in descending order, and the top N with the highest similarity is taken as the output recommendation to users. The reason (class label) of the recommended landscape and the input case is the same, which indicates that the recommended landscape is similar to the input case. In addition, the case process and judgment results in the recommended garden landscape are similar to the input cases, indicating that the results of garden landscape recommendation based on multimodal features are more accurate.

### 3.2. Deep Neural Network Node Connection

By training a deep neural network node interface with a known dataset, a backpropagation algorithm can be used to make the network score learn to extract information for classification while discarding irrelevant information. For example, when distinguishing round and square color blocks, the shape of the color block edge is the information used for classification, and the color of the color block itself is irrelevant. For another example, when training a neural network classifier, if the garden images of each class in the training set are taken in both bright light and low light, the neural network will focus on those features that are not related to brightness. Therefore, the neural network interface can be used as a feature extractor in many occasions, which can convert high-dimensional landscape images into low-dimensional feature maps.

(3) $$\begin{array}{c} \text{CenterMath}\left(x,y\right)=\frac{1}{n-1}\times \frac{\sum \left[x\left(t,j\right),y\left(j-1\right)\right]}{\sum_{i,j<t}x\left(i,j\right)-y\left(i-1\right)}-\frac{1}{\sum n\left(x,y\right)-1}. \end{array}$$

In order to verify the effectiveness of the multimodal feature-based garden landscape recommendation method proposed in this paper, a series of comparative experiments are also carried out using Word2vec features, TF-IDF features, Word2vec features, and TF-IDF features of garden landscapes in series. Features and high-level fusion features of Word2vec features and TF-IDF features are used for referee document recommendation. When the recommended number N is 10, 30, and 50, respectively, the accuracy rate, recall rate, and F1 value of garden landscape recommendation are compared.

(4) $$\begin{array}{c} \frac{p\left(x,x\left(t\right)\right)}{\sum p\left(x\right)-p\left(t\right)}\cap \frac{q\left(t,t\left(x\right)\right)}{\sum q\left(x\right)-q\left(t\right)}=\mathbb{Z}\left(x,t\right). \end{array}$$

From the comparison of accuracy results, it can be seen that the accuracy of garden landscape recommendation using direct series features (w2v + tfidf) is slightly higher than that of single-modal Word2vec feature recommendation (w2v), indicating that multimodal features perform better than single-modal features. However, when N is 30 or 50, the accuracy of garden landscape recommendation using concatenated features (w2v + tfidf) is lower than that of using TF-IDF features in Table 1. The recommendation effect of simple concatenation of modal features is not ideal.

The whole network consists of 3 subnetworks: Top.Net, Left.Net, and Front.Net. The original input to the network is 115 × 250 × K, and different transposes are done before feeding into the three networks. For Front.Net, processing is not needed, the network is inputted directly. The network interface layer and the pooling layer of the three subnetworks have the same parameter settings, but due to the different sizes of the input garden video blocks of the three subnetworks, the outputs of the same network interface layer of the three subnetworks are different. In order to further screen the features of the CSP feature matrix in the feature screening stage, the convolution is not performed in the dimension of electrodes here, but only in the dimension of feature points.

### 3.3. Evaluation of Intelligent Computing Accuracy

The design of objects based on multimodal intelligent calculation points mainly includes methods based on global features and matching methods based on local features. A typical method based on global 3D features has a view feature histogram, which can be used to directly calculate the 3D features of a point cloud. Since this feature is based on the whole point cloud, the interesting part of the point cloud needs to be segmented before calculation. Local-based 3D feature classes are similar to local features of 2D landscape images, and then matched with points in known models.

(5) $$\begin{array}{c} \text{Petecell}\left(p,c\left(i,j\right)\right)=\langle \begin{array}{c} \frac{\nabla p\left(i,j\right)\times \nabla c\left(i\right)}{c\left(t\right)}\\ \nabla c\left(i,j\right)-1 \end{array}. \end{array}$$

HOG is used to count the gradient information of the pixels in the garden landscape image. Its main steps are first, multiple pixels in the garden landscape image are formed into a cell, the gradient information of each cell is calculated, then multiple cells are formed into a block, and the gradient histogram of a block is generated. Finally, the gradients of all blocks are combined. The histograms are combined and contrast normalized to obtain the final HOG features. When extracting the HOG of the garden video, first calculate the HOG feature of each garden landscape image in the garden landscape image sequence, and then concatenate the HOG along the time dimension to generate the HOG feature of the garden video. HOG is a local feature descriptor in the air domain, and Laser extracts local features in garden videos in order to better.

**Figure 1:**
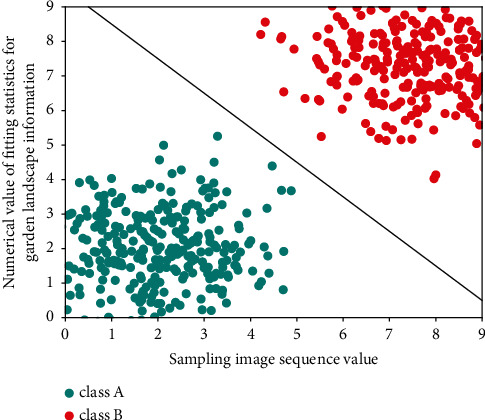
The fitting statistics of garden landscape gradient information.

Usually, the problems of time garden sequence or state garden sequence in Figure 1, such as text prediction, garden image design, and action classification, can be solved by using hidden Markov model (HMM). Moreover, there are two types of data in such problems, one is observable called observation garden sequence, the other is unobservable called hidden garden sequence, and the changes of both garden sequences are random processes. HMM predicts the output of the hidden state garden sequence by observing the probability matrix and state transition matrix of the garden sequence on the basis of the observable sequence.

### 3.4. Deep Neural Network Feature Extraction

The deep neural network interface operation extracts a local information in the garden landscape image, which constitutes a new two-dimensional matrix *k*, which is called the feature map of the garden landscape image under the network score operation network interface operation. Different network scores play different roles. It is a 400 × 400 single-channel grayscale garden image, and the network interface is carried out in an extended way through 4 × 4 cores.

(6) $$\begin{array}{c} \left\{\begin{array}{c} \text{covtion}\left(x,y-1\right)+\text{covtion}\left(x,y\right)>1\\ \text{covtion}\left(x,y\right)-\text{covtion}\left(x-1,y\right)<1 \end{array}\right). \end{array}$$

The network score (a) is the average network score, which blurs the garden landscape image to a certain extent. The network score (b) makes the garden image overexposed. The network score (c) extracts the image of the garden image. This type of network interface operation is the basis of various filter operations commonly used in various garden landscape image processing software. Adjusting the size of the network score, the value of the elements, and the sliding method of the network score on the garden landscape image, are various garden landscape image processing effects that can be obtained.

**Figure 2:**
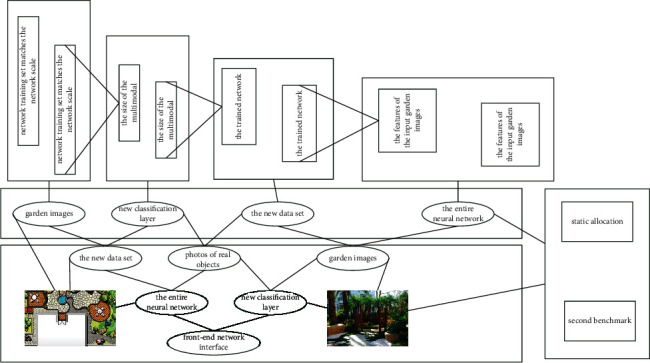
Static allocation of deep neural network features.

The first benchmark (BS1) is derived from a static allocation based approach. After the prediction model in Figure 2 estimates the number of virtual machines needed to complete the task, the model adds an additional 10% of computing resources to avoid the task not being completed within the deadline. For comparison, the second benchmark (BS2) in this experiment comes from the same allocation, but uses 30% more computing resources. The third benchmark (BS3) is derived from a dynamic model, but uses only parameters related to the task run settings for prediction of completion times. The last is the function of this system after concrete realization. More extensive performance tests were performed in this experiment, and the final results are shown in the text.

## 4. Construction of Landscape Design System Based on Multimodal Intelligent Computing and Deep Neural Network

### 4.1. Multimodal Intelligent Computing Analytical Solution

**Figure 3:**
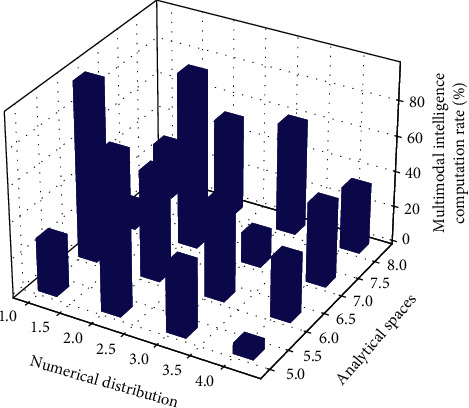
Analytical spatial distribution of multimodal intelligent computing.

It can be seen that the cost of this system to complete multimodal intelligent computing tasks is close to that of BS1, but it can provide a performance similar to that of BS2 in guaranteeing task deadlines. Compared with BS3, the performance of this system is better than that of BS3 in terms of the cost of running tasks and the timeout time of running tasks. Taking the cost and timeout of word count computing tasks as an example, this system only uses 6.64% more cost than BS1, but the average timeout time and the number of tasks that exceed the deadline in Figure 3 are less than the results of BS1.

In general, only using Word2vec features of garden landscapes has the worst recommendation effect, with an accuracy rate of only about 70%, indicating that only using a single modal feature for garden landscape recommendation is not ideal, and there is still a lot of room for improvement. The accuracy of garden landscape recommendation using Word2vec features and TF-IDF features (w2v + tfidf) is slightly higher than the recommendation results using only Word2vec features (w2v), but lower than the recommendation results using only TF-IDF features, indicating that the effect of using multimodal features for landscape recommendation is better than that of using only single-modal features, but the multimodal features of garden landscape cannot be simply connected in series.

### 4.2. Factor Analysis of Landscape Design

An optimal public space filter is created, which can maximize the variance of one type and minimize the variance of the other type. By diagonalizing the covariance matrix of the two types of tasks at the same time, the characteristics of the maximum resolving power of the two tasks are obtained. Relatively speaking, the scale of the fully connected layer of the VGG-16 network is too large, which not only takes up too much storage and computing resources, but also leads to overfitting. Among them, all network interface layers are left as-is. The pooling layer after the last network interface layer is increased to 6 × 6, which reduces the resolution of the output feature map from 7 × 7 to 3 × 3. The size of subsequent fully connected layers is also greatly reduced. The number of parameters of the VGG-16 fully connected layer is about 120 × 106. After modification, if the final output vector is 50 dimensions, the parameters are reduced to about 5 × 106.

(7) $$\begin{array}{c} \arg\min\left\{\sum de^{q}\left(t,t-i\right)-\sum dp^{q}\left(i,i-t\right)\right\}-1=0. \end{array}$$

**Figure 4:**
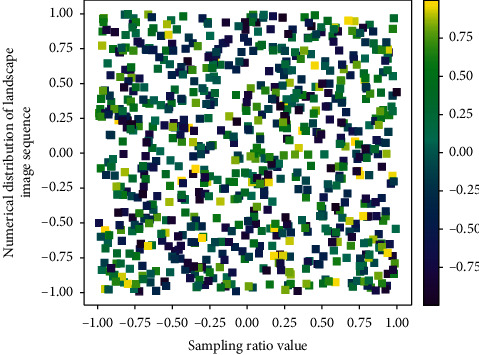
Formatting distribution of garden landscape image sequence.

Compared with the garden video, the garden landscape image sequence increases the time dimension, so the depth kernel is based on the 2D network score to increase the time dimension, and then the depth of the garden video block is used to extract the garden landscape image sequence or garden. The original garden landscape image sequence or garden video passes through the depth to generate a feature cube (the 2D network interface generates a feature map), and the feature cube then goes through the multimodal convolution layer as shown in Figure 4 to extract spatiotemporal features to generate a new feature cube.

The performance of this system is similar to that of BS2 in this performance index. There will be tasks that exceed the deadline mainly because of the prediction error in the prediction model. A more detailed explanation is that when the completion time of the original task only slightly exceeds the deadline, the prediction model in the elastic resource management module will have a hard time deciding whether to perform an operation that needs to be expanded, so the time for expansion will be too late, and the completion time exceeds the deadline.

### 4.3. Deep Neural Network Data Cleaning

In practice, in order to effectively operate objects according to the characteristics of different deep neural networks, information such as the precise shape and orientation of the object is required. Methods using machine learning can extract this information from 2D garden landscape images, but these methods require a large amount of training data. A more direct approach is used to compute this information directly from the multimodal intelligence computing point. Multimodal intelligent computing points are discrete samples of continuous object surfaces, and the effectiveness of point cloud-based algorithms depends on the quality of the samples.

(8) $$\begin{array}{c} \int f\left(x,t\right)\times g\left(x-t\right)\text{d}x\text{d}t-\int g\left(x,t\right)\times f\left(t-x\right)\text{d}x\text{d}t=0. \end{array}$$

A single three-dimensional garden sequence can only collect the information of a certain side of the object at the same time, which makes the results of various multimodal intelligent calculation point algorithms have great deviations. After calibrating multiple 3D garden sequences, the respective multimodal intelligent calculation points can be merged together to form a complete point cloud of the object.

**Figure 5:**
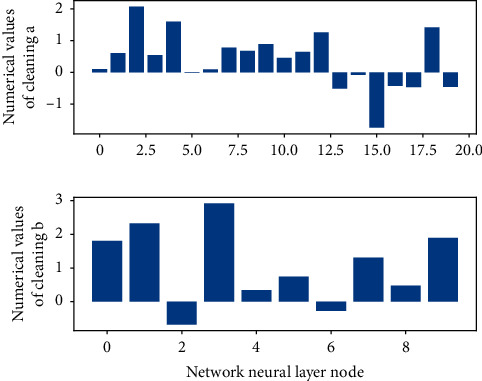
Deep neural network data cleaning statistics.

In the first type of dynamic garden landscape garden video, the largest has 113 frames of garden landscape images, and the smallest has 13 frames of garden landscape images, so even with the same garden landscape, the frame difference contained in the garden data in Figure 5 is still very large. In order to better extract the spatiotemporal features of dynamic garden landscapes, we first extract key frames with a uniform frame number for all garden video samples. The key frame extraction in garden video is mainly used in garden video retrieval, by extracting the most representative one frame of garden landscape image or multiple frames of garden landscape image to replace the whole garden video.

(9) $$\begin{array}{c} \left\{\begin{array}{c} \lim\left[f\left(x,t-1\right)-\text{d}x^{q}\left(x-t,t\right)\right]>0,\\ \lim\left[x\left(t,t-1\right)\right]>1. \end{array}\right) \end{array}$$

The key frame extraction of garden video based on motion information mainly extracts the key frames shown in Table 2 according to the change of the moving target in the garden landscape image, and the change of this motion information is reflected in the garden video as the pixels between adjacent garden landscape images. The optical flow method obtains the target motion information by calculating the temporal change of the corresponding pixels of two adjacent frames in the garden video stream. The feature matrix is sent to CNN for learning, and then the weights of its fully connected layer are extracted, and the distribution of its weights is used to determine which parts of the feature matrix are more effective for classification, so as to screen some features.

The multimodal network score slides in a garden video block to interface with the corresponding small cube, and finally generates a feature cube, thus ensuring the spatiotemporal information of the original input. Note that the original garden video block time dimension-3 is larger than the depth kernel time dimension. If the time dimension of the garden video block is equal to the time dimension of the depth kernel, at this time, the plane feature map is generated after the network interface, and the spatiotemporal information will be quickly lost after a depth operation, so this situation is generally placed in the last network interface. Layers are used to synthesize spatiotemporal information.

## 5. Application and Analysis of Landscape Design System Based on Multimodal Intelligent Computing and Deep Neural Network

### 5.1. Multimodal Intelligent Computing Data Preprocessing

The incremental relationship of pixels in the garden image that requires multimodal intelligent computing is not preserved, but the symmetrical features on both sides of the diagonal line are still preserved. If the network score is slid one pixel at a time, the result size of the extended network interface operation is the same as the original garden landscape image size. At the same time, it can be seen that the network interface results at this time already include the results of the network interface in the aforementioned feasible methods, which are marked with bold fonts in the figure, and the results in the edge part are distorted to a certain extent.

(10) $$\begin{array}{c} \left(\begin{array}{cc} \sin\;x & -1\\ 1 & \cos\;x \end{array}\right)\left(\begin{array}{cc} \frac{1}{x} & -1\\ 1 & \frac{-1}{x} \end{array}\right)=\left(\begin{array}{cc} f\left(x\right)g\left(x-t\right) & 1\\ 1 & g\left(x\right)f\left(x-t\right) \end{array}\right). \end{array}$$

**Figure 6:**
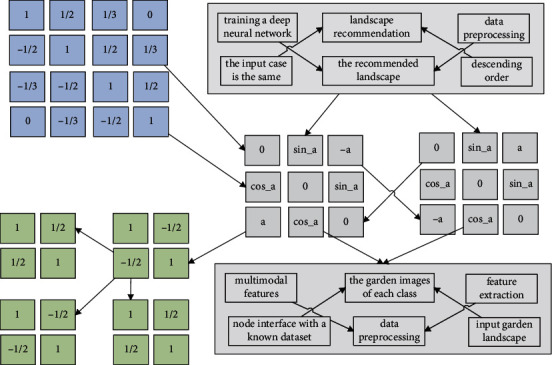
Multimodal intelligent computing data convolution.

The size of the result is easier to calculate when the network interface is performed in this way, so it is more commonly used in the neural network interface model of Figure 6. Because only the edge pixels are distorted to a certain extent in the final result, this effect can be ignored when the size of the garden image is large. Then the normalized features are input into the network in series, the output neurons of each layer in the network can be calculated by formula, all layers adopt this calculation method, and each neuron is connected with all neurons in the previous layer, The weight of each feature value is automatically learned, and different weights can find the complementary relationship between the RGB feature and the depth feature. When using deep neural network to analyze the network weights, the weights of the fully connected layer are usually used, and the analysis of the convolution kernel weights also has certain significance.

According to the motion information *M* of each frame, the key frames of the garden video can be extracted. Suppose we extract K frames as key frames. At this time, we sort the motion information *M*, and then select the garden videos corresponding to the first K M. The frame is the key frame of the garden video. If the total number of frames in a garden video is *T* < *K*, the key frame extraction will not be performed on the garden video, and the random nearest neighbor interpolation method is used to expand the total number of frames in the garden video to K frames. We can find out the parts that are beneficial to classification by observing the weight matrix, so as to filter and optimize the original feature matrix through the analysis of the weights, and obtain a more effective feature set.

(11) $$\begin{array}{c} \left[\begin{array}{cc} i-x\left(i\right) & i+x\left(i\right) \end{array}\right]\left[\begin{array}{c} x\left(i\right)\\ x\left(1-i\right) \end{array}\right]=\left[\begin{array}{cc} 1 & 0\\ 0 & -1 \end{array}\right]. \end{array}$$

The data used in the previous experiments were all obtained by simulating the network to compute MapReduce tasks in a multimodal network, so these data samples lacked the setting of the deadline. This section uses the results of the initial resource recommendation model corresponding to the number of resources running these tasks as the deadlines for these tasks, because the result of the initial resource recommendation model is the most reasonable estimate of the completion time of the task without the performance degradation of the task during the calculation process.

### 5.2. Simulation Realization of Garden Landscape Design

This garden landscape design experiment shows a MapReduce task with obvious performance degradation. During the running process, the computing speed of the task and the average CPU utilization of the virtual machines in the cluster change with time. Around 200 seconds, the performance of the virtual machine running this task dropped. However, CPU utilization did not change much. The reason for this phenomenon is that CPU utilization alone cannot be used as a criterion for evaluating whether a virtual machine performance degradation has occurred.

(12) $$\begin{array}{c} G\left(p,i,j\right)=\left\{\begin{array}{c} \underset{i,j<p}{\sum }\frac{p\left(a\right)+p\left(b\right)}{\ln\left(i+xj\right)}\\ \underset{i,j<p}{\sum }\frac{p\left(a+b\right)}{\ln\;p\left(i+xj\right)} \end{array}\right). \end{array}$$

**Figure 7:**
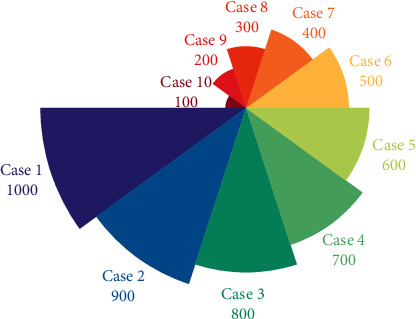
Neural network interface weight distribution.

In the first two loops of training, in order to prevent the network from overfitting, the learning rate is set to 0.1 and 0.3, respectively. This method is called the “warmup” of network training. In the third loop, the learning rate is set to 0.61. The subsequent loop dynamically adjusts the learning rate according to the performance of the current model on the test dataset. When the test accuracy of Figure 7 reaches 75%, the learning rate is set to 0.5; when the accuracy reaches 85%, it is set to 0.251. Due to the great difference between the two images, the extracted features have different value ranges in terms of value. If they are simply spliced and then put into the fully connected layer, the network needs to automatically adjust the parameters to adapt to this range difference. It may reduce the learning efficiency and final performance of the network. Therefore, we use normalization to normalize both features to a certain range, where the value is between −1 and 1.

**Figure 8:**
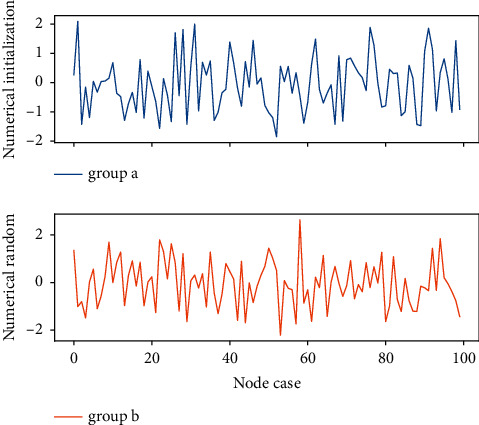
Random initialization distribution of multimodal data.

After training, the neural network interface will generate a network weight model, which stores the value of the network score in each network interface layer in the neural network interface. Before training, the network score of each layer is the values that are initialized randomly. As the training progresses, the value of the network score keeps approaching a certain feature extractor. After the training, the value of these network scores is saved as the weight model of the network. Note that at this time, the weight model in Figure 8 is only suitable for designing multimodal data, and the data of other modes cannot be designed or the design rate is very low.

Decoding is also very efficient by using each output node of the DNN to estimate the posterior probability of a certain state of a continuous density HMM given the observed features of a garden image and trained using the Viterbi algorithm. In a deep neural network, triphones are usually bound as a clustering state, which is used as an output unit of the neural network to replace the monophone state. The advantages of this are first, the DNN-HMM system is implemented with minimal modifications to the existing multimodal neural network system. Second, the DNN output unit can directly reflect the performance improvement.

### 5.3. Example Application and Analysis

When training multimodal neural network data, the goal in the garden image design system is to minimize the empirical risk in the sense of joint probability, which involves linguistically labeling garden sequences and extracting garden image features at frame level. In the large-vocabulary garden image design system designed in this paper, word-level tags are used instead of state-level tags. Parameter binding is often used as a normalization method when training ASR systems based on multimodal neural networks. It can be seen that the accuracy of using the RGB image is higher than that of the depth image in both the seen case and the unseen case, which is obvious because the RGB image contains more information.

When a network has finished learning a certain type of data, observe its weights, and you can see that the larger weights are useful weights for classification, while the smaller weights are weights that are not useful for classification. Horizontally, the accuracy obtained in the case of using only RGB images is higher than that in the case of unseen, but the accuracy obtained by using the depth image is just the opposite, and the accuracy in the case of seen is lower than that in the case of unseen. It can be seen that the spatial geometric features represented by the depth image will have a great auxiliary role in classifying the unseen instances in the training set in the same class, but the disadvantage is that for the instances that already exist in the training set, when testing is easy to get confused.

(13) $$\begin{array}{c} \langle )\begin{array}{c} \min\text{teration}\left(q,p\right)=\left\{q\left(r-1\right),p\left(r-1\right),f\left(q⟶p\right),g\left(q⟶p\right)\right\}\\ \max\text{teration}\left(q,p\right)=\left\{q\left(r\right),p\left(r\right),u\left(q⟶p\right),v\left(q⟶p\right)\right\} \end{array}. \end{array}$$

When solving the problem of garden sequence design, it is often solved by finding the minimum cost, in WFST by finding the maximum or minimum weight path. The state weights after the transition are all prepended. Doing so gradually removes redundant paths, reducing the overall search time. The weights are prepended, the total weight of each path will not change, and finally minimization is done. The prediction model based on Mode 1 can only achieve satisfactory prediction results on the validation data set with a small gap. However, on a validation set with a large gap, the resulting prediction error will be very large. The prediction model based on Modality-2 only achieved poor prediction performance on all types of validation sets. However, the model's predictions did not vary much across all types of validation sets.

**Figure 9:**
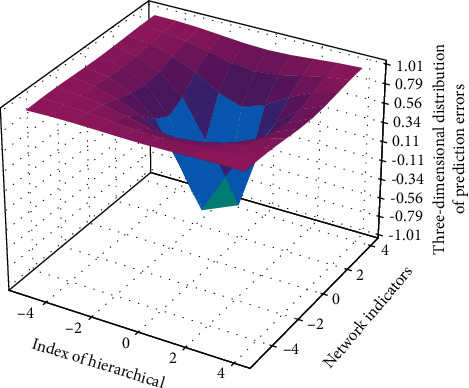
Three-dimensional distribution of prediction error of multimodal network.

The prediction model based on Mode-3 cannot be successfully trained. During the training process in Figure 9, the training error has not been able to converge. Finally, the multimodal-based prediction model achieved very good prediction results on all validation sets. The main function of determinization is to keep the one with the smallest weight when the transition probability of jumping out of a state is equal to the input probability of entering this state. In this way, the transition probability of each state is determined without affecting the overall result. For each input, there is a unique deterministic output. In order to make the value adaptable, we choose a number slightly larger than the average value 700, that is, the number of frames of all samples is normalized to 700 frames, that is, the dimensions of all frame features are normalized to 700. For statistical features, each sample is the same value, and we also extend it to 700 dimensions. This shows that the method in this paper can screen out the most effective features.

## 6. Conclusion

Based on multimodal intelligent computing and deep neural network theory, this paper constructs a static garden landscape design model in space, and completes the process of transforming garden landscapes in sequence into dynamic garden landscapes. The simulation implementation of dynamic garden landscape design based on multimodal intelligent computing points is divided into two categories: one is low-level dynamic garden landscape design, including garden landscape segmentation, garden landscape tracking, garden landscape feature extraction, and garden landscape classification. The requirements for the algorithm are very strict at each stage. The other is the dynamic garden landscape design based on the neural network interface. This method only needs to send the garden landscape image sequence into the designed network structure to obtain the classification result directly. The design process is simple and accurate. In this context, this paper selects the multimodal depth-based dynamic garden landscape based on neural network interface for research and analysis and uses small-scale garden images to conduct experiments in the simulation process to compare multimodal features and pure low-noise features in different noise environments. We can try more feature extraction methods and carry out corresponding feature optimization, from which analysis can deepen the understanding of network behavior and draw conclusions about network learning behavior. The experimental results show that the multimodal garden image model based on deep neural network reduces the recognition error rate of garden details, and in practical applications, the actual garden details can be realized by extracting the above 20 modal features and restore the system and reduce the 130-mode features extracted in large quantities to 20 modes, which can effectively reduce the complexity of the design system.

## Figures and Tables

**Table 1:** Simple series description of multimodal features.

Simple type	Input number	Series description	Output number ratio/%
TF-cov1	16 × 16 × 16	The different sizes	30.412
TF-cov2	32 × 32 × 32	The outputs of it	27.516
TF-cov3	64 × 64 × 64	The three subnetworks	23.108
TF-cov4	128 × 128 × 128	The same network	30.412
IDF-pool1	16 × 16 × 16	Interface layer	18.311
IDF-pool2	32 × 32 × 32	The input garden	32.279
IDF-pool3	64 × 64 × 64	The three subnetworks,	24.782
IDF-pool4	128 × 128 × 128	Of video blocks	9.641

**Table 2:** Garden landscape image extraction key frame mode.

Extraction key frame model	Garden landscape image factor
The optical flow method	Bullet.draw(graphics);
Bullet = bulletlist.get(i);	By calculating the *s*^2^+*t*^2^
Motion information *v*(*i*) > 1	Plane.keyrelasedcontroldirection(e);
Bulletlist.remove (*i*);	Pixels of two adjacent frames *p*(*x*, *x*(*t*))
The key frame *i* > 1	Gamestate = false;
Public void keyreleased(keyevent e)	In the garden video stream *v*(1, *i*)
The key frames in *q*(*x*) − *q*(*t*)	If (bulletlist.size() = = 0){
Mainly extracts exp(*c*(*i*)	Ax.set_xlabel('Numerical distribution')
Based on *h*(1, *i*)	Ax.set_ylabel('Analytical spaces')
Extraction of *hq*(*t*+1)	Game.loadgame();
Of the corresponding *m*+*δ*(*q*(*t*))	Obtains the target motion information
Public static void main(String args) {	Temporal change *dM*^*q*^(*t*)

## Data Availability

The data used to support the findings of this study are available from the corresponding author upon request.
